# Modulation of type 2 inflammation during grass pollen-specific sublingual immunotherapy

**DOI:** 10.3389/fimmu.2026.1784244

**Published:** 2026-03-09

**Authors:** Lorenzo Salvati, Manuela Capone, Alessio Mazzoni, Anna Vanni, Giulia Lamacchia, Cristina Scaletti, Paola Parronchi, Francesco Liotta, Francesco Annunziato, Lorenzo Cosmi, Laura Maggi

**Affiliations:** 1 Department of Clinical and Experimental Medicine, University of Florence, Florence, Italy; 2 Immunology and Cell Therapy Unit, Careggi University Hospital, Florence, Italy; 3 Flow Cytometry Diagnostic Center and Immunotherapy, Careggi University Hospital, Florence, Italy; 4 Immunoallergology Unit, Careggi University Hospital, Florence, Italy

**Keywords:** AIT, ILC2, SLIT, sublingual grass immunotherapy, Th2 cells, type 2 inflammation

## Abstract

**Introduction:**

Both ILC2 and Th2 cell subsets play a critical role as functional effector cells in the pathogenesis of allergic diseases. Although allergen-specific immunotherapy is currently the only disease-modifying therapy available for allergic disorders, the immunological mechanisms interfering with type 2 immune response are not yet fully explored.

**Methods:**

This study focuses on describing the immunological changes caused by standardized grass pollen-specific sublingual immunotherapy (SLIT) in a cohort of patients with seasonal allergic rhinitis. Patients diagnosed with moderate to severe seasonal allergic rhinitis were enrolled and treated with grass pollen-specific SLIT for a duration of three years. We investigated circulating CD4+ T cells and ILC2 via flow-cytometry, assessing their cytokine expression. Grass-specific IgE levels were assessed.

**Results:**

We observed a decrease of frequencies of IL-4 and IL-13 producing, and CD154-expressing, CD4+ T cells after one year of treatment, while frequencies of IFN-γ producing CD4+ T remained stable. We also observed significant and long-term clinical improvement induced by SLIT, although grass-specific IgE levels remained relatively stable over time.

**Discussion:**

These exploratory findings collectively suggest early modulation of the type 2 immune response with sustained clinical response regardless of persistent allergic sensitization in patients undergoing grass pollen-specific SLIT for allergic rhinitis.

## Introduction

Type 2 immune response is critical in the pathogenesis of allergic diseases and it is characterized by a close collaboration between innate and adaptive immune responses including both type 2 innate lymphoid cells (ILC2) and Th2 cell subsets. In the case of allergic rhinitis, the allergic sensitization initiates at the nasal mucosa by disrupting the epithelial barrier (1). The release of epithelial-derived cytokines, such as TSLP, IL-25, and IL-33, originating from the epithelium along with the production of chemokines, including CCL20, attracts dendritic cells to the site of inflammation (2). These mediators promote the differentiation of immature dendritic cells and activate ILC2, which subsequently release IL-5 and IL-13 (3). Dendritic cells then migrate to the draining lymph nodes, where they present the antigen and promote the differentiation of naïve T cells into Th2 effector cells and follicular T helper cells (Tfh), further promoting the inflammatory process (4). IL-4 drives the maturation of B lymphocytes into plasmablasts and plasma cells, which produce IgE (5). IgE binds to the high affinity receptor (FcϵRI) expressed on mast cells and basophils (6). Allergen-specific immunotherapy (AIT) is the sole disease-modifying therapy currently available for treating allergic disorders (7, 8).

Subcutaneous immunotherapy (SCIT) and sublingual immunotherapy (SLIT) effectively reduce mast cell and basophil degranulation (9, 10). AIT involves high-dose allergen exposure, which prompts dendritic cells to express regulatory markers, decrease CD86 expression, and produce IL-10 and IL-12 (11). This fosters T cell anergy and the formation of regulatory T cells (Treg), inhibiting the Th2 response. In addition to suppressing the Th2 response, Treg cells also promote a shift toward a Th1-oriented response during AIT (12). In house dust mite-specific SLIT, after six months, a reduction in Der p 1-specific T cell proliferation along with increased IFN-γ and IL-10 production, has been observed (12). AIT influences allergen-specific antibody production: SCIT induces IgG (especially IgG4), while SLIT boosts IgA, blocking IgE-allergen complexes and preventing mast cell and basophil degranulation, hence reducing allergy symptoms (7, 13–16). During the type 2 immune response, IgE-allergen complexes bind to the low-affinity IgE receptor (FcϵRII, also known as CD23) on the surface of B cells, leading to cross-linking of the receptors and influencing IgE production and antigen presentation. Inhibiting this pathway stops B cells from presenting allergens to T cells, impeding the B cell-mediated Th2 response. In long-term tolerance induced by AIT, IL-10-producing regulatory B cells (Breg) and allergen-neutralizing antibodies play key roles (17, 18).

In patients with grass pollen-induced allergic rhinitis, SLIT has been shown to improve clinical symptoms and has lasting effects that extend beyond the treatment period (19, 20). ILC2 play a role in driving the induction and maintenance of allergic inflammation at mucosal surfaces (21, 22). In previous reports, it was observed that SLIT-treated patients did not exhibit the seasonal increase of circulating ILC2, differently from patients who didn’t undergo AIT (23). Moreover, during grass pollen-specific SLIT, a distinct population of IL-10-producing ILC2 has been observed, and its presence has shown a correlation with the clinical efficacy of SLIT (24). Additional data have indicated the potential role of ILC2 modulation towards a regulatory phenotype also in food allergy (25).

In the present study, patients diagnosed with seasonal allergic rhinitis who tested positive in skin prick tests for grass pollen extract were included. These patients underwent SQ-standardized sublingual immunotherapy for a treatment period of 3 years. The evolution of symptoms was assessed. ILC2 and Th2 cells were analyzed from peripheral blood samples to evaluate the modulation of the type 2 immune response.

## Methods

### Patients

Adult patients with clinical history of moderate to severe grass pollen-induced allergic rhinitis (interfering with usual daily activities or sleep), a positive skin prick test to grass pollen extract, who did not respond to symptomatic treatment and received grass pollen-specific SLIT (Grazax^®^ - Phleum Pratense, 75000 SQ-T/2800 BAU, ALK-Abellò A/S, Hoersholm, Denmark), were enrolled at Careggi University Hospital, Firenze, Italy. Skin prick test was performed by pricking a drop of grass pollen extract (grass mix, Lofarma, Italy) on the volar surface of the forearm. Positive skin prick test was defined when the diameter of the grass pollen-induced wheal was ≥ 3mm or ≥ than the diameter of the histamine-induced wheal. Exclusion criteria were defined in agreement with clinical contraindications to allergen immunotherapy suggested by EAACI position paper (26). All patients were followed up to 3 years from the initiation of treatment (1 tablet per day for 36 consecutive months). The evolution of symptoms was assessed using a visual analogue scale (VAS), ranging from 0 (no symptoms) to 10 (worst symptoms ever), which patients completed annually. Blood samples were collected at T0 (before starting SLIT), at T1 (one year after), at T2 (two years after) and at T3 (three years after the initiation of SLIT).

### Grass-specific IgE assessment

IgE serum levels were measured by ImmunoCAP^®^ assay (Phadia 250, Thermo Fisher Scientific, Uppsala, Sweden). Values are expressed as KUA/L.

### Immunophenotyping by flow cytometry

Mononuclear cells (MNC) were obtained from peripheral blood (PB) by means of centrifugation on Ficoll-Hypaque gradient. PBMNC were stained for 15 minutes with fluorochrome-conjugated mAbs (**Supplementary Table 1**) to identify ILC2 and Th2 cells. The gating strategy used to identify ILC2 and Th2 cells is reported in **Supplementary Figure 1**. To evaluate cytokines production by flow cytometry, PBMNCs were then polyclonally stimulated for 6 hours with PMA (10 ng/mL) and ionomycin (1 μM), the last 4 hours in the presence of brefeldin A (5 μg/mL). Cells were then fixed in formaldehyde 2% for 15 minutes at room temperature, washed in PBS plus BSA 0.5%, and then intracellularly stained with fluorochrome-conjugated mAbs (listed in **Supplementary Table 2**) in the presence of the permeabilizing agent saponin (0.5%). Samples were acquired on a BD LSR II flow cytometer (BD Biosciences). Evaluations were performed at T0 (before SLIT), T1 (one year), T2 (two years), T3 (three years) after the initiation of therapy.

### Statistics

ANOVA with Bonferroni *post-hoc* test was used for multiple comparisons. For two-group comparisons, T-test was used. In all tests, a value of p ≤ 0.05 was considered significant. Data were analyzed using OriginPro, Version 95E (OriginLab Corporation, Northampton, MA, USA).

## Results

### Long-term clinical improvement and stable grass-specific IgE levels in patients treated with grass pollen SLIT

**Figure 1:**
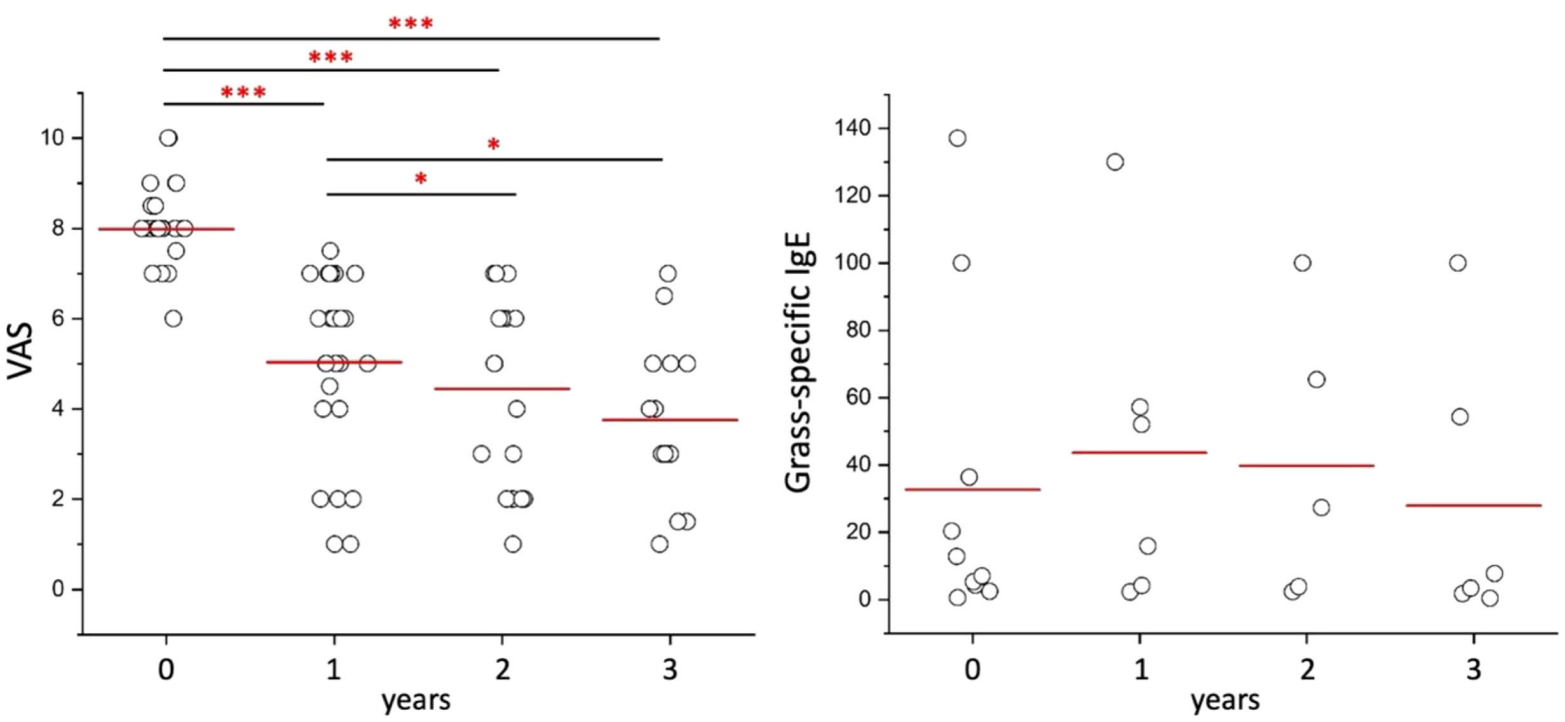
Longitudinal evaluation of clinical symptoms and IgE levels during grass pollen-specific sublingual immunotherapy at baseline, 1, 2 and 3 years after the initiation of treatment. Panel A shows Visual Analogue Scale (VAS) scores available for 28 patients at T0, 26 patients at T1, 18 patients at T2, and 14 patients at T3. Panel B shows grass-specific IgE levels available for 10 patients at T0, 6 patients at T1, 5 patients at T2, and 6 patients at T3. Each dot represents a single patient. Red lines indicate the mean values. ***p<0.001, *p<0.05 calculated with one-way ANOVA and Bonferroni *post-hoc* test.

A total of 28 patients (18 males and 10 females, mean age 36,2 ± 13,8 years) with seasonal allergic rhinitis and positive results on skin prick tests for grass pollen were enrolled in the study. The majority of patients were polysensitized to inhaled allergens (75%, 21/28 cases). A total of 10 (35.7%) had concomitant mild asthma. None had atopic dermatitis. The majority of patients were of Caucasian origin, with only one from North Africa. At baseline, the mean VAS was (mean ± SD) 8,0 ± 0,9 indicating the relevant burden of symptoms experienced by the patients in their daily lives. A significant clinical improvement was demonstrated as early as one year after the initiation of grass-specific SLIT (mean 5,0 ± 2,0) (**Figure 1A**). This improvement in symptoms persisted, with a further significant reduction of VAS at 2-year follow-up (mean 4,4 ± 2,1) followed by a stabilization at the 3-year follow-up (mean 3,8 ± 1,8) assessments (**Figure 1A**). However, in contrast to clinical improvement, the levels of grass-specific IgE remained relatively stable over time, as illustrated in **Figure 1B**. Grass-specific IgE levels (mean ± SD) were 32,6 ± 47,3 KUA/L at T0 compared to 27,9 ± 40,8 KUA/L at T3 (**Figure 1B**).

### Frequencies of circulating CD4+ T cells producing type 2 cytokines decrease already after one year of SLIT

**Figure 2:**
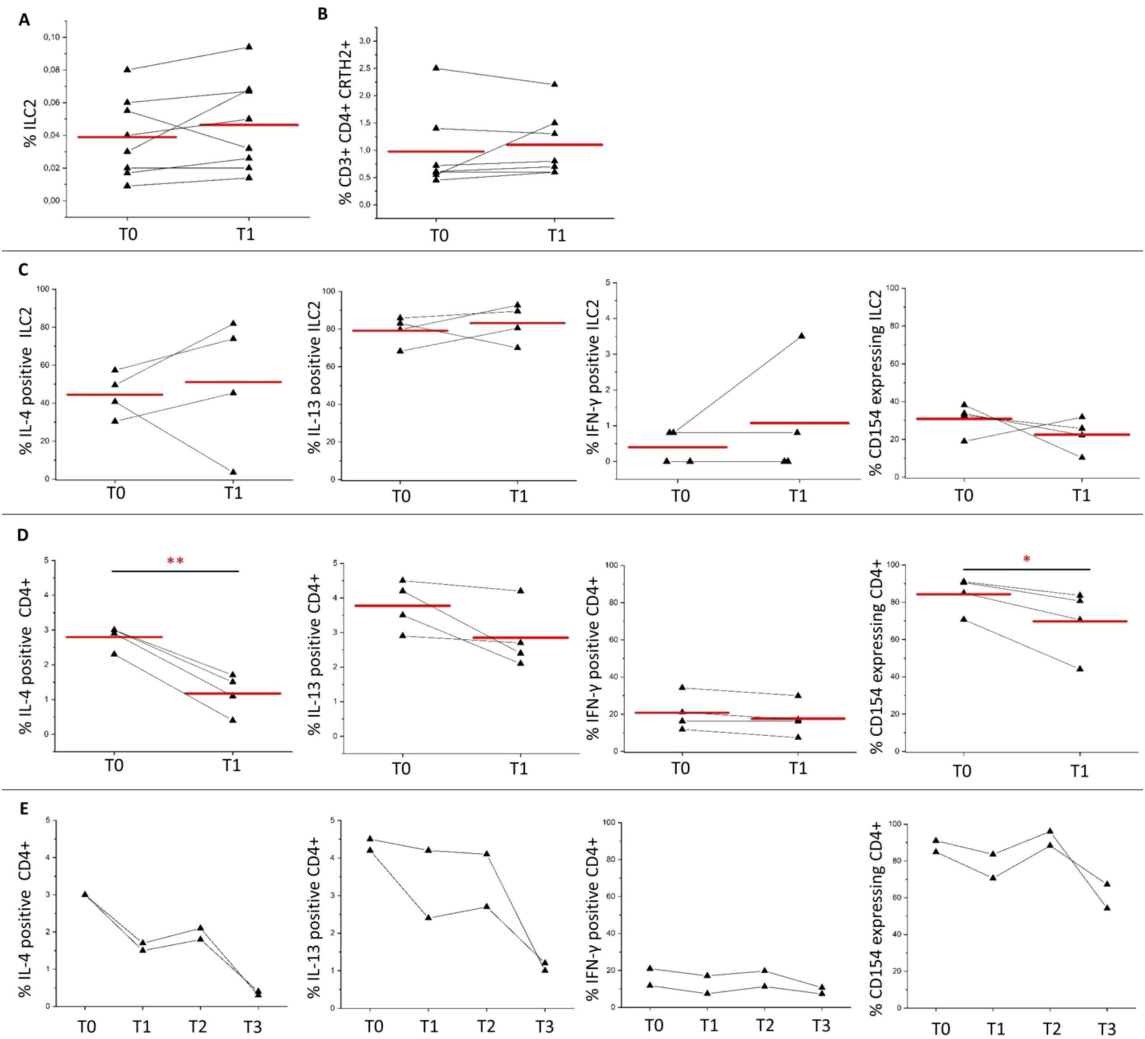
ILC2 and CD4+ T cells response induced by grass pollen-specific sublingual immunotherapy. **(A)** shows percentages of ILC2 in 8 patients (6 males, 2 females) at baseline (T0) and 1 year after SLIT initiation (T1). **(B)** shows percentages of Th2 cells in 7 patients (5 males, 2 females) at baseline (T0) and 1 year after SLIT initiation (T1). **(C)** shows percentages of ILC2 producing IL-4, IL-13, IFN-γ, or expressing CD154 in 4 patients (2 males, 2 females) after T0 (before starting SLIT) and T1 (1 year after the initiation of SLIT). **(D)** shows percentages of IL-4, IL-13 or IFN-γ producing CD4+ T cells and of CD154 expressing CD4+ T cells in 4 patients after *in vitro* polyclonal stimulation at T0 and T1. **(E)** shows longitudinal evaluation of percentages of IL-4, IL-13 or IFN-γ producing CD4+ T cells and of CD154 expressing CD4+ T cells at T0 (before starting SLIT), T1 (1 year), T2 (2 years), and T3 (3 years after the initiation of SLIT) in 2 patients. Red lines in **(A–D)** indicate mean values. * p<0.05, ** p<0.01 calculated with two-tailed paired T-test.

After one year of SLIT, percentages of both ILC2 and Th2 cells remained stable in 8 patients (6 males and 2 females) examined, as shown in **Figures 2A, B**. To investigate the cytokine production, in a subset of 4 patients (2 males and 2 females) we examined the intracellular levels of IL-4, IL-13, and IFN-γ after *in vitro* PMA/Iono stimulation. Interestingly, we found that there were no significant differences in the expression of these cytokines or of CD154 in ILC2 cells after one year of SLIT, as shown in **Figure 2C**. However, in contrast, there was a reduction of the frequencies of IL-4 positive, IL-13 positive and CD154-expressing CD4+ T cells (**Figure 2D**), whereas the frequency of IFN-γ positive CD4+ T cells was stable at one-year follow-up (**Figure 2D**). The long-term longitudinal analysis of 2 patients who exhibited 70% and 25% improvement in VAS scores respectively, confirmed the trends towards reduction of IL-4, IL-13, and CD154-expressing CD4+ T cells at the 3-year evaluation compared to baseline (**Figure 2E**). Percentage of IFN-γ positive cells was stable over the years (**Figure 2E**).

### Different prevalent cytokine production by lineage-CD161+ cells after one year of SLIT might reflect different clinical response

**Figure 3:**
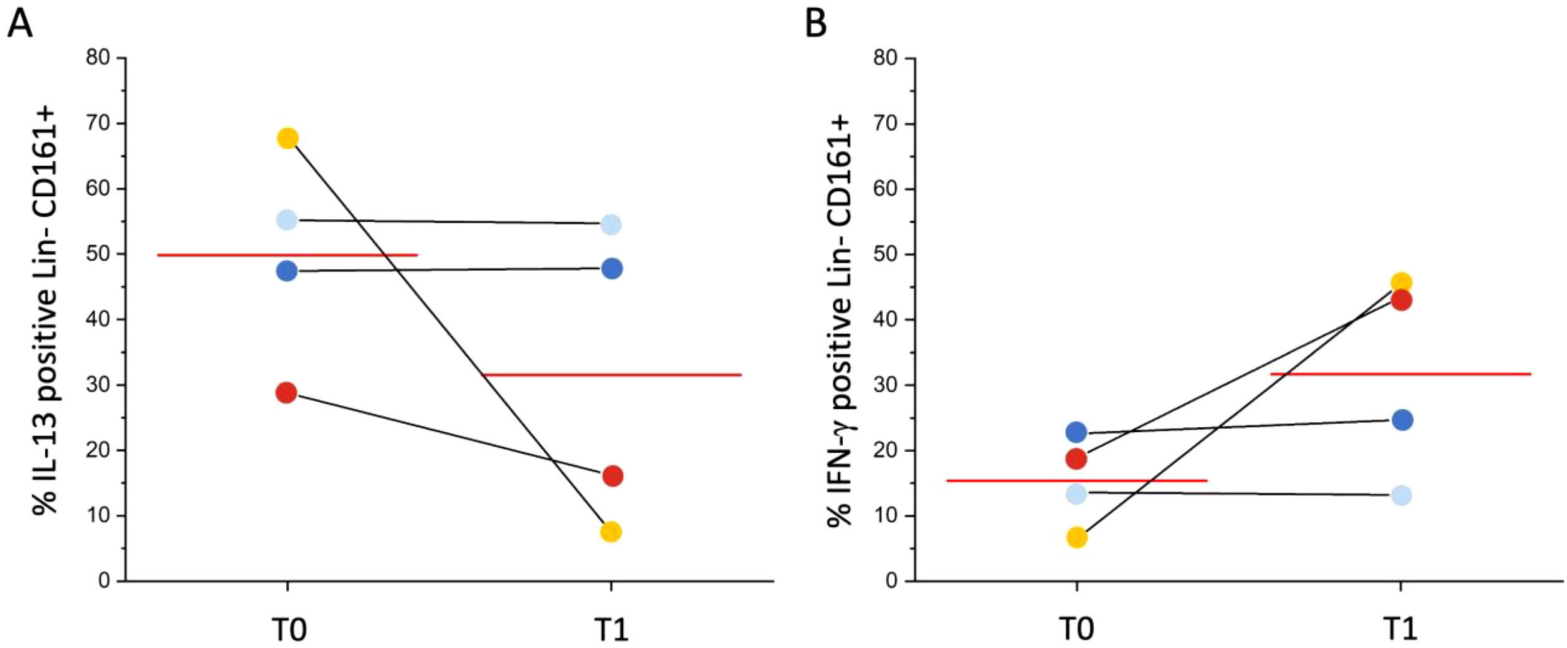
Longitudinal evaluation of IL-13 or IFN-γ positive Lin-CD161+ cells. **(A)** shows percentages of IL-13-positive Lin-CD161+ cells at T0 (before starting SLIT) and T1 (1 year after the initiation of SLIT) in 4 patients, after *in vitro* polyclonal stimulation; **(B)** shows percentages of IFN-γ-positive Lin-CD161+ cells at T0 (before starting SLIT) and T1 (1 year after the initiation of SLIT) in 4 patients, after *in vitro* polyclonal stimulation. Each color identifies a distinct patient: yellow and red dots represent patients with higher clinical improvement (53% VAS reduction); whereas light blue and dark blue dots represent patients with lower improvement (29% VAS reduction). Red line indicates mean value.

Expression of IL-13 or IFN-γ after *in vitro* polyclonal stimulation were also evaluated on Lineage-CD161+ cells in the same 4 patients. No significant differences in cytokine production were observed at one-year follow-up as shown in **Figures 3A, B**. However, patients reporting an average 53% improvement in VAS score (yellow and red dots, **Figures 3A, B**) showed a decrease of the frequency of IL-13 positive Lin-CD161+ cells and increase of the frequency of IFN-γ positive Lin-CD161+ cells, while patients reporting a lower clinical improvement (average 29%, light blue and dark blue dots, **Figures 3A, B**) maintained almost stable both IL-13 positive and IFN-γ positive Lin-CD161+ cells’ percentages at one year follow-up.

## Discussion

This study aimed to investigate type 2-immunological changes in patients with seasonal allergic rhinitis who underwent grass pollen SLIT in association with the long-term clinical improvement. The improvement in symptoms started just after 1-year treatment and maintained up to three-year follow-up confirms the long-lasting effectiveness of grass pollen SLIT in alleviating the symptoms associated with seasonal allergic rhinitis. Notably, after 3 years of treatment good control of symptoms was obtained with quite interindividual variability. The use of VAS score, which is a global patient-reported measure, can be intrinsically subjective, although it correlates with nasal symptom severity (27, 28). The use of different symptom scales, in different studies, could explain the small benefit of the grass pollen SLIT in reducing symptoms reported in a previous meta-analysis (29). A limitation of our study is that VAS scores were exclusively assessed and no additional clinical scores were available. On the other hand, the levels of grass-specific IgEs remained relatively stable over time, as previously shown (30). Durham and colleagues previously demonstrated increases in grass pollen-specific serum IgG4 and IgE-blocking factor levels in SLIT-treated patients (15, 29). Compared to grass-specific IgE routinely measured, as in our study, the presence of IgE-blocking components can be evaluated using a functional assay that measures the amount of IgE actually hindered from binding to the allergen (15, 31, 32). The consistent levels of grass-specific IgEs suggest that while SLIT effectively managed the clinical symptoms, it did not significantly impact the overall production of grass pollen-specific IgEs, although a significant increase of IgE-blocking factors is known to occur during treatment (31). IgE-blocking factor’s ability is to interfere with the binding process between IgEs and their receptors, thus preventing the activation of mast cells and basophils.

One year of SLIT resulted in stable ILC2 and Th2 cells’ percentages. The analysis of cytokine production in a subset of patients with seasonal allergic rhinitis undergoing SLIT revealed that the modulation of the type 2 immune response in patients treated with grass pollen SLIT involves an early reduction of IL-4 and IL-13 CD4+ T cells and CD154-expressing CD4+ T cells. On the contrary, no apparent early cytokine production changes were observed in ILC2 population. After one year of continuous treatment, there were no significant differences in the production of IL-4, IL-13, IFN-γ, or the expression of CD154 in ILC2 population. Similarly, IL-13 or IFN-γ positive Lin-CD161+ cells were not significantly changed at one-year follow-up, although patients with rapid relevant clinical improvement exhibited decrease of IL-13 positive Lin-CD161+ cells and increase of IFN-γ positive Lin-CD161+ cells. This observation altogether with the stable cytokine production by ILC2 at one-year follow-up, might suggest the relative increase of other ILC subsets, such as ILC1-like cells producing IFN-γ (33, 34). Moreover, a significant reduction of CD4+ T cells expressing IL-4, a cytokine central to type 2 immune responses, was observed. Comprehensively, these exploratory data indicate a downregulation of type 2 inflammation. Additionally, CD154-expressing CD4+ T cells decreased. These results suggest the modulation of T-cell response towards a less activated phenotype specifically induced by SLIT. CD4+ T cell-response changes observed at the 3-year longitudinal evaluation in SLIT-treated patients with clinical improvement further support the long-term effectiveness of the immune-modulation induced by SLIT, although derived from a very limited number of patients. The main limitation of this exploratory study is the small sample size and the lack of a sample size assessment, this is due to monocentric, observational and prospective nature of the study. However, even if other study limitations should be taken into consideration (i.e., lack of a control group, and potential confounders, as environmental factors), a strength point of this study is the paired-longitudinal analysis of the same patients over time allowing to obtain relevant results also with a small number of enrolled patients.

In conclusion, the study highlights the long-term clinical improvement achieved with grass pollen-specific SLIT in patients with seasonal allergic rhinitis. While the levels of grass-specific IgEs remained stable, indicating a persistence of allergic sensitization, SLIT effectively modulated type 2 immune response towards a less activated phenotype. These findings contribute to our understanding of the immunological changes induced by SLIT and support its potential as a therapeutic approach for allergic diseases. Indeed, despite its proven effectiveness, the use of AIT is largely undervalued in clinical practice, and only a small percentage of patients who would benefit from the therapeutic effects of AIT are offered this option (35). The knowledge of the mechanisms by which AIT works, may be of help in increasing AIT use in clinical practice with lasting health benefits for an ever-increasing number of patients.

## Data Availability

The original contributions presented in the study are included in the article/**Supplementary Material**. Further inquiries can be directed to the corresponding author.
