# Supplementary material for: Modulation of type 2 inflammation during grass pollen-specific sublingual immunotherapy
Source: Front Immunol. 2026 Mar 9;17:1784244. doi: 10.3389/fimmu.2026.1784244 (PMC13006293; doi:10.3389/fimmu.2026.1784244)

**Supplementary Table 1.** List of all fluorochrome-conjugated mAbs used for flow cytometric immunophenotyping of ILC2 and Th2 cells.

| **Antigen** | **Fluorochrome** | **Clone** | **Company** |
| --- | --- | --- | --- |
| Lineage-1* | FITC | multiple | BD Biosciences |
| CD15 | FITC | MMA | BD Biosciences |
| CD123 | FITC | 7G3 | BD Pharmingen |
| TCRαβ | FITC | WT31 | BD Biosciences |
| CRTH2 | PE | BM16 | Miltenyi Biotec |
| CD161 | APC | DX12 | Miltenyi Biotec |
| CD45 | PerCP | 2D1 | BD Biosciences |
| CD4 | PE-Cy7 | SK3 | BD Biosciences |
| CD3 | Pacific Blue | UCHT1 | BD Biosciences |

* Lin-1 cocktail includes CD3 (SK7), CD14 (MφP9), CD16 (3G8), CD19 (SJ25C1), CD20 (L27), CD56 (NCAM16.2)

**Supplementary Table 2.** List of all fluorochrome-conjugated mAbs used for intracytoplasmatic staining of ILC2 and Th2 cells.

| **Antigen** | **Fluorochrome** | **Clone** | **Company** |
| --- | --- | --- | --- |
| Lineage-1* | FITC | Multiple* | BD Biosciences |
| CD15 | FITC | MMA | BD Biosciences |
| CD123 | FITC | 7G3 | BD Pharmingen |
| TCRαβ | FITC | WT31 | BD Biosciences |
| CRTH2 | PE | BM16 | Miltenyi Biotec |
| IL-13 | APC | JES-105A2 | BD Biosciences |
| CD45 | PerCP | 2D1 | BD Biosciences |
| CD161 | PE-Cy7 | 191B8 | BD Pharmingen |
| IFN-γ | Pacific Blue | B27 | BioLegend |
| IL-4 | APC-Cy7 | MP4-25D2 | BioLegend |
|  |  | | |
| Lineage-1* | FITC | multiple | BD Biosciences |
| CD15 | FITC | MMA | BD Biosciences |
| CD123 | FITC | 7G3 | BD Pharmingen |
| TCRαβ | FITC | WT31 | BD Biosciences |
| CRTH2 | PE | BM16 | Miltenyi Biotec |
| CD45 | PerCP | 2D1 | BD Biosciences |
| CD161 | PE-Cy7 | 191B8 | BD Pharmingen |
| CD154 (CD40L) | APC | TRAP1 | BioLegend |
| IL-4 | APC-Cy7 | MP4-25D2 | BioLegend |
|  |  | | |
| CD4 | PE-Cy7 | SK3 | BD Biosciences |
| CD8 | PerCP | SK1 | BD Biosciences |
| CD154 (CD40L) | PE | TRAP1 | BD Biosciences |
| IFN-γ | FITC | 25723.11 | BD Biosciences |
| IL-13 | APC | JES-105A2 | BD Biosciences |
| CD3 | Pacific Blue | UCHT1 | BD Biosciences |
| IL-4 | APC-Cy7 | MP4-25D2 | BioLegend |

* Lin-1 cocktail includes CD3 (SK7), CD14 (MφP9), CD16 (3G8), CD19 (SJ25C1), CD20 (L27), CD56 (NCAM16.2)

**Supplementary** **Figure 1.** Gating strategy used to identify ILC2 and Th2 cells among peripheral blood mononuclear cells (PBMNCs). P1, based on physical parameters, allows for the identification of lymphocytes’ islet. P2 identifies CD45+ cells, P3 identifies CD45+Lin-CD123-CD15- cells, and P5 identifies ILC2 (Lin-CD161+CRTH2+). From gate P2, P4 allows to identify CD3+CD4+ T cells and among these, quadrant Q2 identifies Th2 subset (CD3+CD4+CRTH2+). Plots from a representative study patient are depicted.


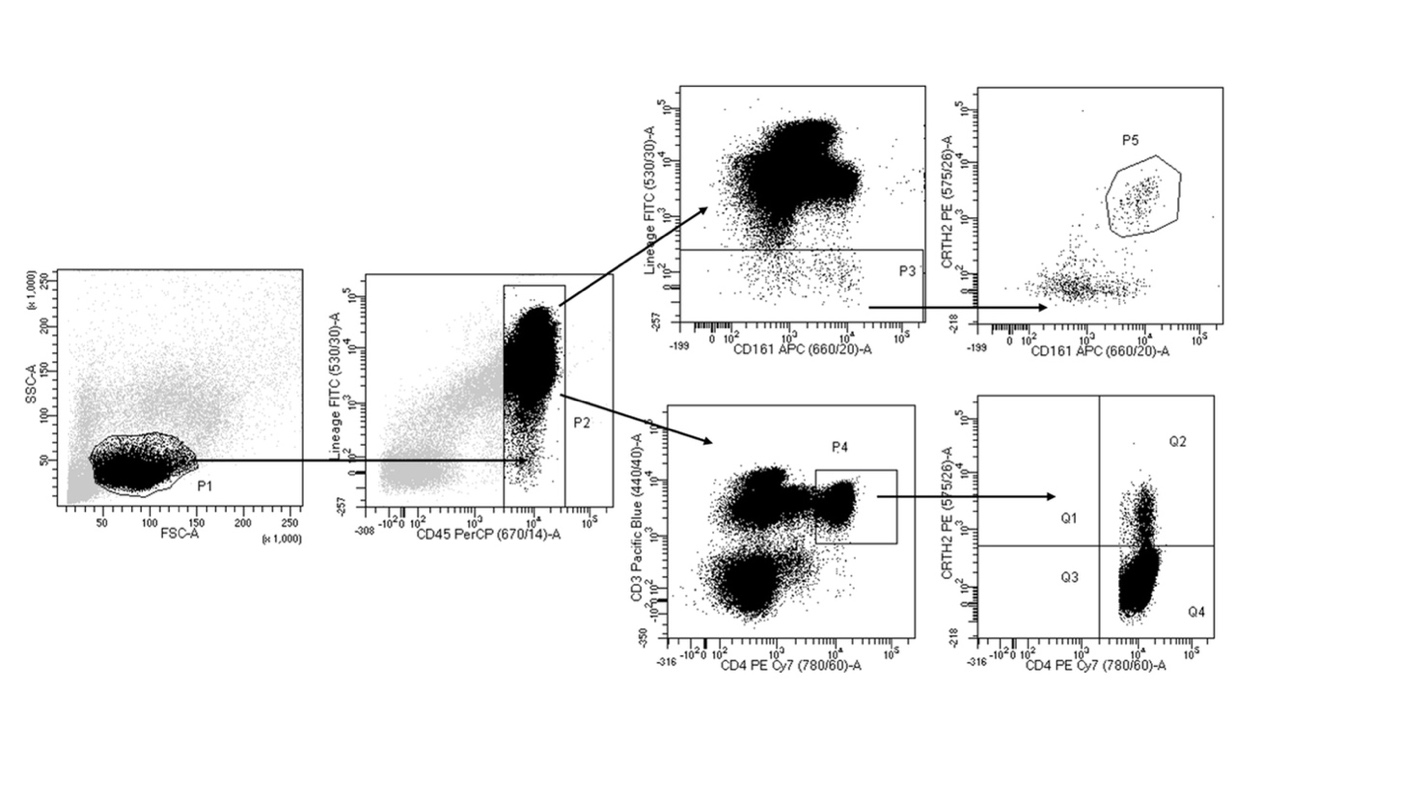

Supplement: Supplementary file 1 [file DataSheet1.docx]
